# Comparison of Nebivolol and Bisoprolol for Cardiovascular Mortality in Hypertensive Patients

**DOI:** 10.7759/cureus.6453

**Published:** 2019-12-23

**Authors:** Ratan Kumar, Kheraj Mal, Jamila Begum, Faizan Shaukat

**Affiliations:** 1 Cardiology, Khairpur Medical College, Nawabshah, PAK; 2 Cardiology, National Institute of Cardiovascular Diseases, Sukkur, PAK; 3 Internal Medicine, Dow University of Health Sciences, Karachi, PAK; 4 Internal Medicine, Jinnah Post Graduate Medical Center, Karachi, PAK

**Keywords:** cardiology, bisoprolol, nebivolol

## Abstract

Introduction

Beta-blockers again are now considered as first-line therapy for various cardiovascular diseases. In this study, we compare the cardiovascular event between two beta-blocker, i.e. Nebivolol and Bisoprolol.

Materials and Methods

It is a two-arm open-label randomized prospective study that was conducted from 1^st^ Jan 2016 to 30^th^ July 2019 in tertiary care hospital, Nawabshah. One thousand and fifty-six (n=1056) hypertensive patients were enrolled after informed consent, which were randomized into two equal groups. Patients were followed up for one year.

Results

Comparison between Nebivolol and Bisoprolol showed that all-cause mortality (9.8% vs 11.48%), cardiovascular mortality (5.4% vs 7.0%), all-cause hospitalization (14.4% vs 16.3%), and cardiovascular hospitalization (9.8% vs 12.09%) was numerically lower in nebivolol but the difference was not statistically significant.

Conclusion

Further large scale multicentric trials with a longer follow up period are needed to compare various beta-blockers for cardiovascular event.

## Introduction

Agents that block the adrenergic β-receptors, beta-blockers, have been a cornerstone for the treatment and management of cardiovascular disease (CVD) for more than a decade. Increase survival rate and expectancy of patients with CVD due to the development of primary prevention and early-detection strategies along with the development of new and effective therapeutic agents has increased the prevalence of cardiovascular disease [1]. In 2013, European Society of Cardiology (ESC) moved beta-blocker to second-line therapy for the treatment of essential hypertension based on various meta-analysis conducted on beta-blockers such as atenolol and propranolol [2-3]. ESC, in 2018, issued its guideline, which suggested beta-blocker as the first line of therapy for indications such as heart failure, hypertension with angina, hypertension with myocardial infarction, coronary artery disease [4]. This change in attitude towards the use of beta-blocker is mainly due to the introduction of a new generation of beta-blockers such as Nebivolol. Various data on Nebivolol have shown that it has increasing favorable effects on central blood pressure, stiffness of the aorta, and endothelial dysfunction. There is no risk of new-onset diabetes, and a more favorable side effect profile compared to conventional beta-blockers [4]. Bisoprolol, carvedilol, and nebivolol have shown to improve outcomes in RCTs in heart failure [5]. Despite being in use in Pakistan for decades, there is limited data available regarding cardiovascular protection offered by beta-blockers. In this study, we will compare the two most commonly used beta-blockers (Nebivolol and Bisoprolol) for the cardiovascular outcome.

## Materials and methods

This two-arm open-label randomized prospective study was conducted from 1st Jan 2016 to 30th July 2019 in tertiary care hospital, Nawabshah. One thousand and fifty-six (n=1056) hypertensive patients were enrolled after informed consent, which were randomized by a 1:1 ratio using an online software research randomizer. Group A was given standard hypertensive therapy (angiotensin-converting enzyme inhibitors, angiotensin receptor blockers, calcium channel blocker or diuretics) and Nebivolol. Group B was given standard hypertensive therapy and Bisoprolol. Patient’s characteristics such as age, gender, history of smoking, duration of hypertension, family history were noted in the self-structured questionnaire. Their standing blood pressure was also recorded. Patients were followed for a minimum of one year for the development of any cardiovascular event. Patients with less than one year of follow up were counted as lost to follow up. Statistical analysis was done using SPSS v. 22.0 (IBM Corporation, Armonk, New York, United States). Continuous variables including age, blood pressure (BP), and duration of hypertension were analyzed via descriptive statistics and were presented as mean and standard deviation (SD) while categorical variables, including gender, smoking history, and cardiovascular outcomes were presented by percentages and frequencies.

## Results

One thousand and fifty-eight (1058) participants were enrolled and randomized into two equal groups; one with Nebivolol and one with Bisoprolol. The characteristics were comparable between the two groups except for BMI. Lost to follow up were 29 and 33 participants for Nebivolol and Bisoprolol, respectively (Table 1).

**Table 1:** Characteristics of Participants AMI: Acute myocardial infarction.

Characteristics	Patients with Nebivolol (n=529)	Patients with Bisoprolol (n=529)	P-value
Age	54 ± 19	55 ± 19	0.58
Male/ Female	301 /228	289/ 240	0.55
Smoking	152	149	0.8
Diabetes	76	71	0.6
BMI	24.56 ± 5.19	23.17 ± 6.09	0.001
Previous history of AMI	17	21	0.58
Family history of AMI	28	26	0.77
Lost to follow up	29	33	0.6

Primary outcomes were noted one year after follow up. There were numerically less events in the Nebivolol group compared to Bisoprolol; however, the result was not statistically significant (Table 2).

**Table 2:** Primary Outcome CV: Cardiovascular

Primary Outcome	Nebivolol (n=500)	Bisoprolol (n=496)	HR	P-Value
All-cause mortality	49 (9.8%)	57 (11.49%)	0.77 (0.53 to 1.11)	0.16
CV mortality	27 (5.4%)	35 (7.0%)	0.76 (0.47 to 1.24)	0.28
All-cause hospitalization	72 (14.4%)	81 (16.3%)	0.88 (0.65 to 1.18)	0.39
CV hospitalization	49 (9.8%)	60 (12.09%)	0.80 (0.56 to 1.15)	0.23

## Discussion

Nebivolol has a unique mechanism of action that defers from other beta-blockers. In addition to cardioselectivity mediated via β1 receptor blockade, Nebivolol also works as a B3 agonist, which induces nitric oxide-mediated vasodilation by stimulating endothelial nitric oxide synthase [6]. Bisoprolol work by blocking the beta-1-receptor [7]. In this study, we compared the cardiovascular outcomes of patients on nebivolol and Bisoprolol. Nebivolol reduced the incidence of cardiovascular events numerically more than Bisoprolol, but there was no statistically significant difference between the two.

Our result echoes the result of Nebivolol, Bisoprolol Multicenter Study (NEBIS), which concluded there is no difference in blood pressure control between nebivolol and Bisoprolol [8]. CARNEBI (Multiparametric comparison of CARvedilol, vs. Nebivolol, vs. Bisoprolol in moderate heart failure) showed improvement in exercise performance (p < 0.0001) with nebivolol and Bisoprolol [9].

Individual trials of both Nebivolol and Bisoprolol have shown that they reduce cardiovascular events. Study of Effects of Nebivolol Intervention on Outcomes and Rehospitalization in Seniors with Heart Failure (SENIORS) showed that nebivolol significantly reduces all-cause mortality or cardiovascular hospitalization compared to placebo (95% CI): 0.86 (0.74-0.99); p = 0.039 [10]. The cardiac insufficiency bisoprolol study (CIBIS-II) showed significant mortality advantages over placebo. All-cause mortality was significantly lower with bisoprolol than on placebo (156 [11.8%] vs. 228 [17.3%] deaths with a hazard ratio of 0.66 [11].

Better cardiovascular protection by Nebivolol can be explained because of its unique mechanism of action and super selectivity. Nebivolol has 321-fold higher affinity for human cardiac beta1-receptors versus beta-2-receptors making it more selective for beta1-receptors than any other agent in its class [12]. Nebivolol is 3.5 times more beta-1-adrenoreceptor selective than bisoprolol [13]. Nebivolol because its action on the B3 receptor releases nitric oxide, which may reverse endothelial dysfunction and hence speculated to reverse atherosclerosis [14]. Nitric oxide acts as an endogenous inhibitor of platelet aggregation in the platelets. Hence, nebivolol inhibits platelet aggregation triggered by adenosine diphosphate and collagen [15]. Nebivolol also inhibits the proliferation of human coronary endothelial cells, aortic smooth muscle cells, and smooth muscle cells via nitric oxide delivery [16].

To the best of its knowledge, it is the first study that has compared the cardiac outcome of patients on Nebivolol and Bisoprolol in Pakistan. However, the study has its own limitation. First, there was a statistical difference in body mass index (BMI) between the two groups. Secondly, the follow-up period was only one year. Hence, long-term results were not noted. There were other confounding factors such as cholesterol level and lifestyle, which was not taken to account.

## Conclusions

In this study, overall mortality, over-all hospitalization, CV mortality, and CV hospitalization even though was numerically better in Nebivolol than Bisoprolol but there was no significant difference between the two. With the advancement of beta-blockers, they are now again becoming an important option in the management of patients with hypertension and other cardiac diseases. It is important to understand the properties and advantages of various beta-blockers so that maximum advantage can be provided to the patients. Further, large-scale multicentric trials are needed to compare various beta-blockers.
